# *Cryptococcus* inositol utilization modulates the host protective immune response during brain infection

**DOI:** 10.1186/s12964-014-0051-0

**Published:** 2014-09-10

**Authors:** Tong-Bao Liu, Selvakumar Subbian, Weihua Pan, Eliseo Eugenin, Jianping Xie, Chaoyang Xue

**Affiliations:** Public Health Research Institute, Rutgers University, Newark, New Jersey USA; Laboratory of Mycobacterial Immunity and Pathogenesis, Rutgers University, Newark, New Jersey USA; Department of Microbiology and Molecular Genetics, Rutgers University, Newark, New Jersey USA; Shanghai Key Laboratory of Molecular Medical Mycology, Department of Dermatology, Changzheng Hospital, Second Military Medical University, Shanghai, China; Institute of Modern Biopharmaceuticals, State Key Laboratory Breeding Base of Eco-Environment and Bio-Resource of the Three Gorges Area, key laboratory of Eco-environment of three gorges reservoir, Ministry of Education, School of Life Sciences, Southwest University, Chongqing, China

**Keywords:** *Cryptococcus neoformans*, Cryptococcal meningoencephalitis, Host immune response, Glucuronoxylomannan, Capsule production, Inositol transporters, Genome-wide transcriptome, Quantitative real-time PCR, Cellular networks, Immune pathways

## Abstract

**Background:**

*Cryptococcus neoformans* is the most common cause of fungal meningitis among individuals with HIV/AIDS, which is uniformly fatal without proper treatment. The underlying mechanism of disease development in the brain that leads to cryptococcal meningoencephalitis remains incompletely understood. We have previously demonstrated that inositol transporters (*ITR*) are required for *Cryptococcus* virulence. The *itr1aΔ itr3cΔ* double mutant of *C. neoformans* was attenuated for virulence in a murine model of intra-cerebral infection; demonstrating that Itr1a and Itr3c are required for full virulence during brain infection, despite a similar growth rate between the mutant and wild type strains in the infected brain.

**Results:**

To understand the immune pathology associated with infection by the *itr1aΔ itr3cΔ* double mutant, we investigated the molecular correlates of host immune response during mouse brain infection. We used genome-wide transcriptome shotgun sequencing (RNA-Seq) and quantitative real-time PCR (qRT-PCR) methods to examine the host gene expression profile in the infected brain. Our results show that compared to the wild type, infection of mouse brains by the mutant leads to significant activation of cellular networks/pathways associated with host protective immunity. Most of the significantly differentially expressed genes (SDEG) are part of immune cell networks such as tumor necrosis factor-alpha (TNF-α) and interferon-gamma (IFN-γ) regulon, indicating that infection by the mutant mounts a stronger host immune response compared to the wild type. Interestingly, a significant reduction in glucuronoxylomannan (GXM) secretion was observed in the *itr1aΔ itr3cΔ* mutant cells, indicating that inositol utilization pathways play a role in capsule production.

**Conclusions:**

Since capsule has been shown to impact the host response during *Cryptococcus*-host interactions, our results suggest that the reduced GXM production may contribute to the increased immune activation in the mutant-infected animals.

**Electronic supplementary material:**

The online version of this article (doi:10.1186/s12964-014-0051-0) contains supplementary material, which is available to authorized users.

## Lay abstract

*Cryptococcus neoformans* is an AIDS-associated human fungal pathogen that often causes lung and brain infection and is the leading cause of fungal meningitis in immunocompromised persons. The underlying mechanism of disease development in the brain that leads to cryptococcal brain infection remains incompletely understood. Our previous studies have demonstrated that importers of sugar compound inositol (*ITR*) are required for *Cryptococcus* virulence. Animal studies using a cryptococcal strain lacking two major *ITR*s (*itr1aΔ itr3cΔ*) demonstrated that *ITR*s are required for full virulence during the brain infection, despite the normal in vivo growth of the mutant strain in the infected mouse brains. To understand the immune pathology associated with the virulence reduction of the *itr1aΔ itr3cΔ* mutant, we investigated the host response during mouse brain infection by examining the host gene expression profile using next generation sequencing techniques. Our study shows that compared to the wild type, infection of mouse brains by the mutant strain leads to significant up-regulation of many host genes involved in host protective immune response. Interestingly, a significant reduction in polysaccharide secretion was observed in the mutant cells, indicating inositol utilization plays a role in cell surface capsule production. Because capsule has been shown to play a role in the host response during *Cryptococcus*-host interactions, our results suggest that the increased immune activation in the mutant-infected animals may be due to the reduced polysaccharide secretion that leads to virulence attenuation.

## Background

*Cryptococcus neoformans* is a fungal pathogen that frequently infects the central nervous system (CNS) to cause life-threatening meningoencephalitis. Cryptococcosis accounts for over 620,000 death annually worldwide [1]. The molecular basis of cryptococcal infection of the CNS is an area of intensive investigation. Multiple fungal and host factors have been identified to play a role in the fungal penetration of the blood brain barrier (BBB) and to cause CNS infection [2-5]. The polysaccharide capsule of *C. neoformans* is a major virulence factor that is associated with the outcome following initial pathogen-host interaction, including BBB crossing and establishing CNS infection [6-8]. In addition, mutagenesis studies have shown that cryptococcal urease and inositol transporters (*ITR)* are required for the full virulence as evidenced by the defect of these mutants in penetrating the BBB and causing CNS infection [2,9-11]. Screening for mutants with attenuated virulence yielded multiple genes that are required for the survival of *Cryptococcus* in the cerebrospinal fluid (CSF), including an ubiquitin-like protein (*Rub1*) and the phosphatidylinositol-4-kinase (*Pik1*) [12]. The Rub1 protein is also related to fungal transmigration into the CNS; mutant possessing a null mutation in the putative Rub1 gene exhibited increased transmigration into the brain [13]. Recently, using a spectral counting method, expression of a broad range of host cell proteins involved in cytoskeleton rearrangement, cellular metabolism, intracellular signaling and inflammation were identified to be up-regulated during penetration of the BBB by *C. neoformans* [14]. Despite these findings, the underlying mechanism of frequent CNS cryptococcosis and the host immune responses during cryptococcal infection remains incompletely understood.

Recently, we have shown that *Cryptococcus* contains an unusually large *ITR* gene family [15,16]. Based on our studies on two major *ITR*, Itr1a and Itr3c, we found that *ITR*s are required for full virulence of *Cryptococcus*, particularly during the fungal penetration of the BBB to cause infection of the CNS [9]. In accordance with the importance of fungal *ITR*s, human and animal brains contain high inositol levels. Thus, the ability of *Cryptococcus* to efficiently acquire and utilize host inositol could be associated with the high rate of CNS cryptococcosis. Transcriptome analysis of *Cryptococcus* cells directly isolated from AIDS patients with cryptococccal meningitis also showed up-regulation of *ITR*s during brain infection [17]. In addition, using a murine intra-cerebral infection model, we have shown that mice infected by the *itr1aΔ itr3cΔ* mutant survive significantly longer, compared to those infected by the wild type [18]. However, *in vivo* growth assays in both murine and rabbit CNS showed that the mutant and wild type *Cryptococcus* had similar growth rates, suggesting that the mutant had normal growth in the brain [9]. Therefore, it remains unknown what caused the virulence attenuation of the *itr1aΔ itr3cΔ* mutant strain.

In this study, we tested the hypothesis that during murine brain infection, the differential host response elicited in the mouse brain infected by the wild type and the *itr1aΔ itr3cΔ* mutant strains leads to a difference in disease outcome. We interrogated the host response during brain infection using genome-wide transcriptional analysis by shotgun RNA-Seq technology. Our results show that compared to the wild type, infection with the *itr1aΔ itr3cΔ* mutant led to significantly up-regulation of genes involved in the host protective immune response in the infected mouse brain. In addition, activation of host destructive networks, such as cell death, which can contribute to exacerbated inflammation and tissue destruction, was noted only in the wild type-infected, compared to mutant-infected mouse brains. We further showed that the altered GXM production by the mutant strain could be a potential causal link for the altered host immune responses. Thus, our study highlights the molecular immunologic correlates of host response against *Cryptococcus* infection, and revealed a potential mechanistic explanation for the role of fungal inositol utilization in the establishment of CNS cryptococcosis.

## Results

### Genome-wide transcriptome of mouse brains infected with wild type or the *itr1aΔ itr3cΔ* double mutant *Cryptococcus*

To test the hypothesis that the virulence attenuation of the *itr1aΔ itr3cΔ* double mutant during mouse brain infection is associated with its ability to elicit different host immune response, we analyzed the genome-wide transcriptome of the mouse brain after infection with either the wild type or the *itr1aΔ itr3cΔ* mutant, using RNA-Seq method. More than 343 Mbp of RNA-Seq data was generated for each wild type - and *itr1aΔ itr3cΔ* mutant-infected samples that contain over 7 million reads. The rates of assembled reads in all samples were over 99.1% that covered about 90% of the genome (Additional file 1: Table S1).

**Figure 1:**
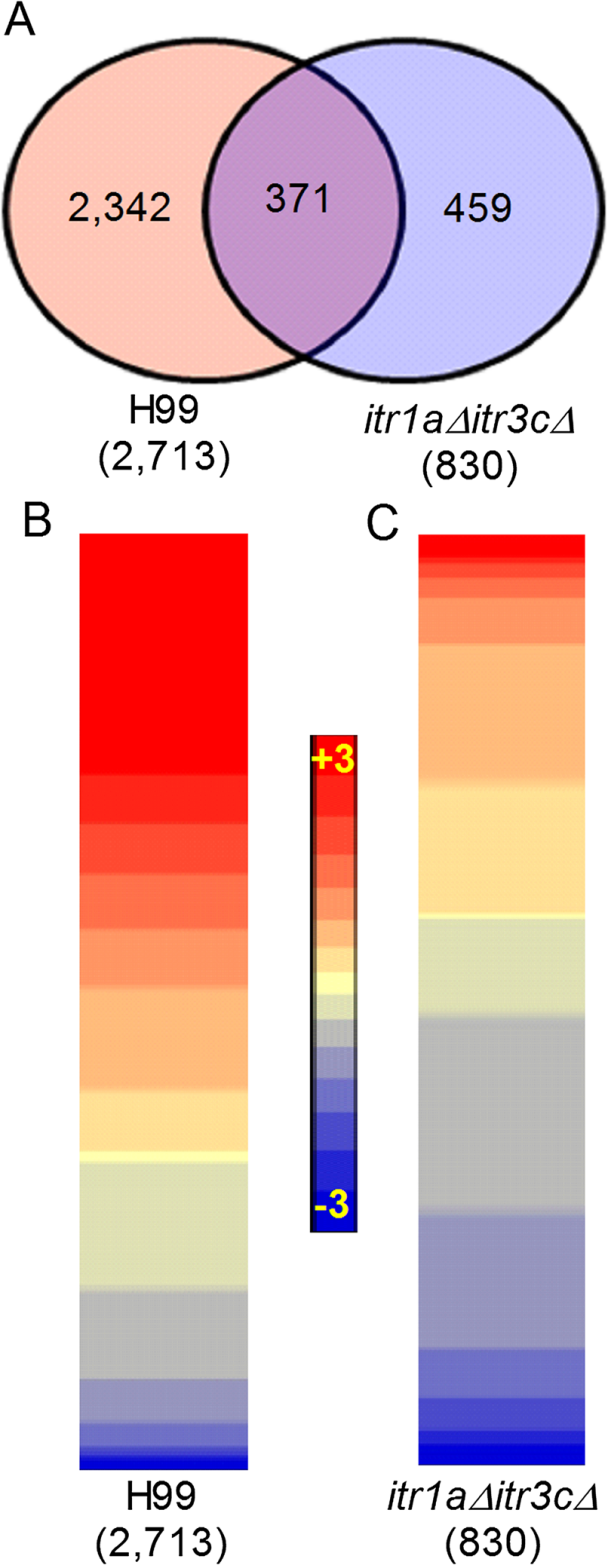
**Summary of genome-wide transcriptome analysis of the wild type or** ***itr1aΔ itr3cΔ*** **mutant infected mouse brain. (A)** Venn diagram showing the number of total and common SDEG dysregulated in the brain of mouse infected with the wild type H99 (red circle) or the *itr1aΔ itr3cΔ* mutant (blue circle). **(B)** Intensity plot of the SDEG from the wild type (H99)-infected, relative to the uninfected, animals. **(C)** Intensity plot of all the SDEG from the mutant-infected, relative to the uninfected, animals. The plots were drawn with descending level of gene expression (top to bottom). The scale bar ranges from +3 (red) to −3(blue) and is common to both (B) and (C). The RNA-seq data was obtained from 3 mouse per group and analyzed group-wise by one-way ANOVA (n = 3/group).

The mapped and annotated RNA-Seq reads were used as input in the Partek Genomics Suite to identify significantly differentially expressed genes (SDEG). We applied a false discovery rate (FDR) of 5% (*q* value ≤ 0.05) as cut-off to select the SDEG from the raw dataset (Figure 1). The density of RNA-Seq reads, taken as equivalent of gene expression, from the wild type- or mutant-infected mouse brain were normalized to the uninfected counterparts. Using this approach, we have identified SDEG that are independently regulated in the wild type or mutant-infected, relative to uninfected mouse brain. Then we compared the differentially expressed genes between wild type- and mutant-infected mouse brains. Using a 5% FDR cut-off, we found 2,713 SDEG (1,113 genes up-regulated; 1,600 genes down-regulated) in the wild type-infected, relative to the uninfected, mouse brain (Figure 1A). In the *itr1aΔ itr3cΔ* mutant-infected mouse brain, we have identified 830 SDEG (552 genes up-regulated; 278 genes down-regulated). Of the 552 up-regulated genes, more than 55% were expressed by greater than 2 fold; while only 22 of the 278 SDEG were down-regulated by 2 fold or more. In contrast, about 12% of SDEG were up-regulated and 13% were down-regulated in the wild type-infected mouse brain (Figure 1B and C). In addition, there were 371 SDEG commonly expressed between the wild type- and mutant -infected brains. In summary, among the total number of SDEG, the expression of about 41% was up-regulated and 59% were down-regulated in the wild type-infected mouse brain; however, about 67% of SDEG were up-regulated and 33% were down-regulated in the mutant-infected mouse brain. Thus, despite of more than 3-fold increase in the total number of SDEG in the wild type-, compared to mutant-infected mouse brain, the percentage of up-regulated genes was greater in the later samples.

### Gene ontology analysis of SDEG

To determine the host biological functions perturbed by the SDEG, we performed gene ontology (GO) analysis using Ingenuity Pathway Analysis (IPA) software with the list of SDEG generated (FDR ≤ 5%) during wild type or mutant infection of mouse brain. The most significantly affected host biological functions were sorted based on the *p*-value significance. As shown in Table 1, the top biological functions that are significantly affected includes neurological disease, host cell death and survival, cellular growth, proliferation and migration, host immune response, cell-cell interaction, nervous system development and function, gene expression, cell movement as well as cell signaling. To understand whether these biological functions were impacted positively (activation) or negatively (inhibition) by the expression pattern of SDEG, we used a z-score algorithm-based prediction from IPA. The z-score algorithm predicts the role of a gene in positively or negatively affecting a biological function based on its expression pattern, relative to the direction and pattern of expression of all member genes that constitute a biological function. Accordingly, among the biological functions commonly shared between the wild type and mutant infections, a significant activation (z-score ≥ 2) in the host cell destruction processes, such as cell death and necrosis, was noted only in the wild type-infected mouse brain. These biological functions were not significantly activated in the mutant-infected brain; in contrast, many SDEG involved in the host protective responses were activated only in the mutant-infected mouse brain. Specifically, the expression pattern of SDEG in these animals was consistent with the activation of proinflammatory response, immune cell trafficking and antimicrobial response, as well as free radical scavenging (Table 1). Taken together, the GO analysis of differential gene expression patterns suggests that activation of biological processes that are detrimental to the host during wild type infection may promote infection and progressive disease pathology. On the other hand, activation of host-protective biological functions associated with reduced inflammation could contribute to effective control of infection and lethality in the mutant-infected animals.

**Table 1:** **Ontology analysis of SDEG in mouse brain infected by wild type or** ***Itr1aΔ itr3cΔ*** **mutant cells**

		***Wild type***		***Itr1aΔ itr3cΔ***	
**No.**	**Category**	**p value (range)**	**Number of genes**	**p value (range)**	**Number of genes**
1	Neurological Disease	5.56E-61-6.96E-05	792	3.59E-37-9.9E-11	326
2	Cell Death and Survival	7.57E-30-1.19E-04	842	2.9E-45-9.82E-11	379
3	Cell-To-Cell Interaction	1.69E-28-1.02E-04	352	3.09E-42-1.3E-10	248
4	Nervous System Development and Function	1.69E-28-7.79E-05	663	2.11E-11-1.07E-10	108
5	Cellular Growth and Proliferation	2.12E-22-1.02E-04	794	1.6E-47-1.02E-10	381
6	Gene Expression	3.8E-19-7E-05	541	1.37E-15-3.02E-11	112
7	Cellular Movement	1.96E-09-1.2E-04	481	1.32E-58-1.3E-10	293
8	Cell Signaling	8.74E-05-8.74E-05	22	7.41E-18-4.05E-12	129
9	Cell-mediated Immune Response	ns		5.81E-17-3.17E-13	42
10	Humoral Immune Response	ns		9.21E-27-1.02E-10	102
11	Inflammatory Response	ns		2.72E-38-1.2E-10	90
12	Immune Cell Trafficking	ns		1.48E-46-1.3E-10	108
13	Antimicrobial Response	ns		2.21E-15-5.49E-12	212
14	Free Radical Scavenging	ns		1.05E-27-8.25E-11	304

ns-not statistically significant.

### Cellular networks associated with host destructive functions are activated in the wild type *Cryptococcus* infected mouse brain

**Figure 2:**
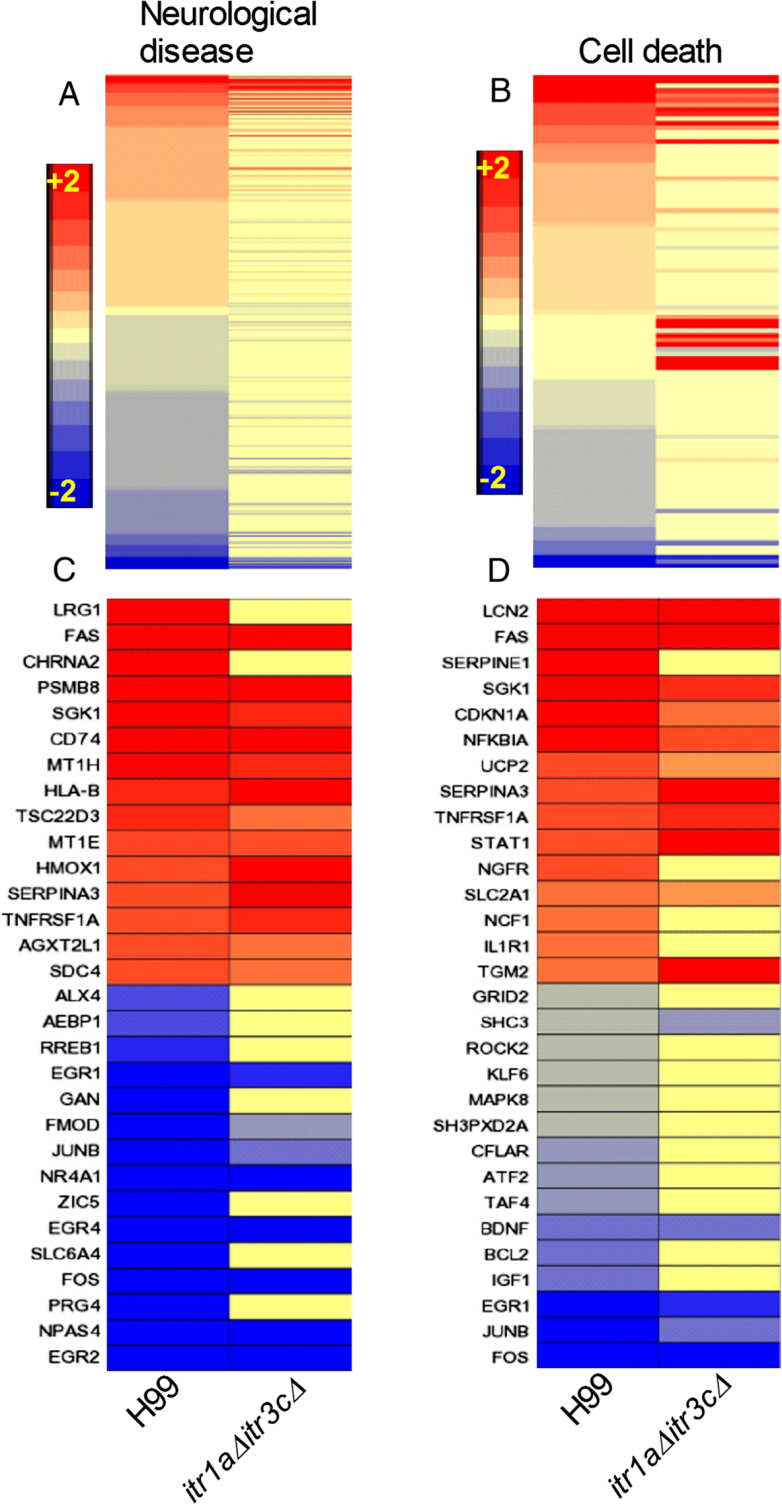
**Expression profile of neurological disease and cell death network genes. (A)** Intensity plot of SDEG (452 genes) involved in neurological disease network in the wild type H99- or the *itr1aΔ itr3cΔ* mutant-infected mouse brain. A subset of 15 most highly differentially expressed genes in this network is listed in **(C)**. **(B)** Intensity plot of SDEG (107 genes) involved in cell death network in the wild type- or mutant-infected animals. A subset of 15 most highly differentially expressed genes in this network is listed in **(D)**. The intensity plots were drawn with descending level of gene expression sorted in the wild type-infected samples (top to bottom). The scale bar ranges from +2 (red) to −2 (blue) and is common to all panels.

Since the GO analysis of SDEG revealed striking differences in the neurological disease and associated cellular functions between the wild type- and mutant-infected mouse brains, we investigated the expression patterns of specific network genes involved in these biological functions. Of the SDEG, a subset of 452 was involved in neurological disease network (Figure 2A, 2C and Additional file 2: Table S2). These network genes code for G protein-coupled receptors (9 genes), enzymes (139 genes), including peptidases, kinases and phosphatases, transcriptional regulators (56 genes), transmembrane receptors (15 genes) and transporters (49 genes) (Additional file 2: Table S2). Of these SDEG, 215 were up-regulated and 237 were down-regulated by more than 2-fold in mouse brain infected with the wild type. However, expression of 58 SDEG was up-regulated and 50 were down-regulated in the mutant-infected mouse brain. Similarly, a subset of 107 SDEG was involved in the cell death network (Figure 2B, 2D and Additional file 2: Table S2). Most of these genes’ products are localized to the cytoplasm (46 genes), while others are localized to the nucleus, plasma membrane or secreted to extracellular space. Of these SDEG, 52 were up-regulated and 43 were down-regulated during wild type infection. These numbers reduced to 25 and 13, respectively, in the mutant-infected animals. In addition, a subset of 69 genes out of 107 was significantly expressed only in the wild type, compared to 12 genes in the mutant-infected animals. Thus, consistent with ontology analysis, a higher number of SDEG involved in the neurological disease and cell death network were up- and down-regulated in the wild type-infected, relative to the mutant-infected, mice brains. Moreover, the expression pattern of these network genes suggests activation of the neurological disease and cell death only in the wild type-infected mouse brain.

### Cellular networks associated with host protective immune response are activated in the *itr1aΔ itr3cΔ* double mutant infected mouse brain

**Figure 3:**
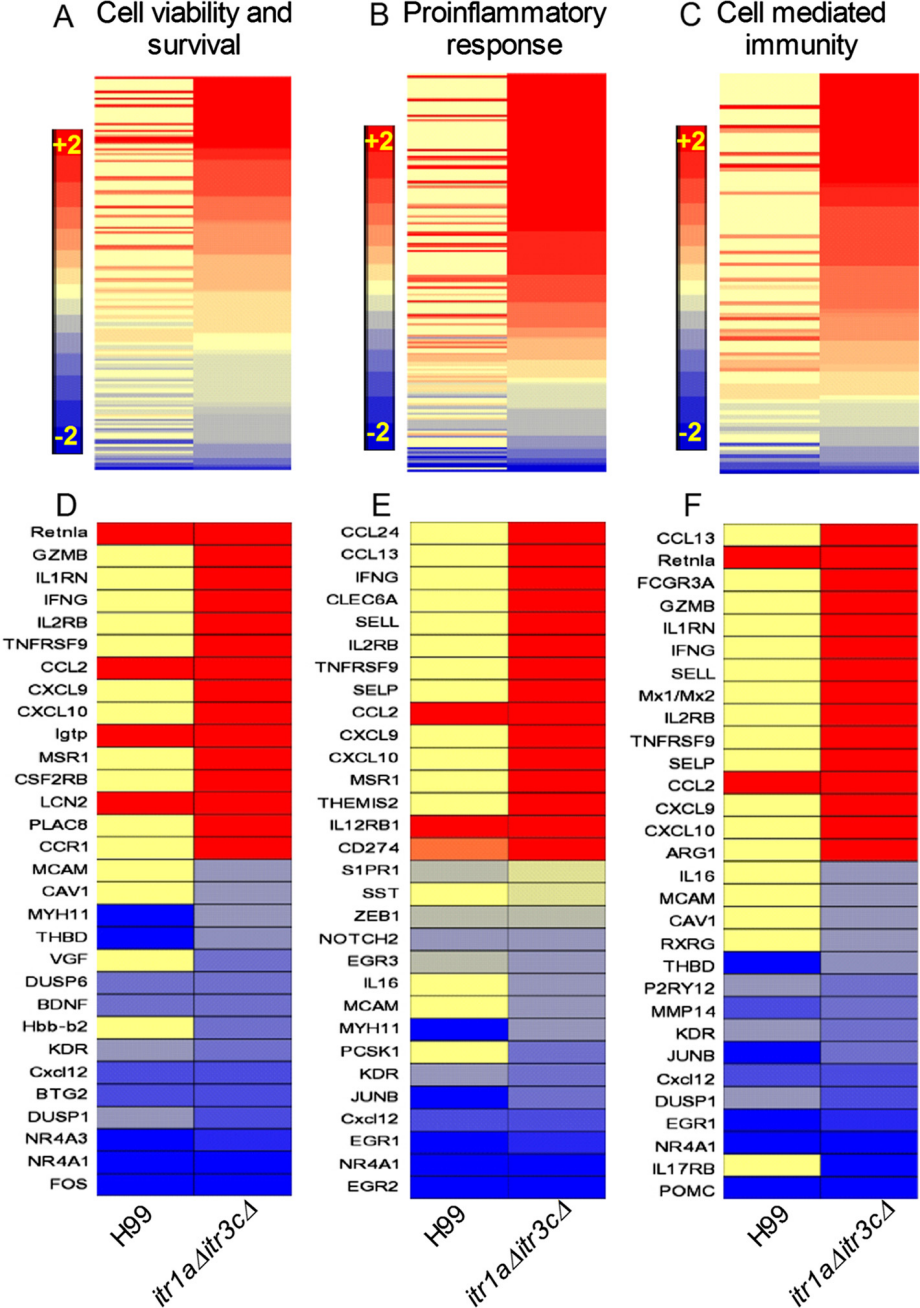
**Expression profile of cell viability and survival, proinflammatory response and cell mediated immune response network genes. (A)** Intensity plot of SDEG (170 genes) involved in cell viability and survival network in the wild type- or mutant-infected animals. A subset of 15 most highly differentially expressed genes in this network is listed in **(D)**. **(B)** Intensity plot of SDEG (174 genes) involved in proinflammatory response network in the wild type- or mutant-infected animals. A subset of 15 most highly differentially expressed genes in this network is listed in **(E)**. **(C)** Intensity plot of SDEG (102 genes) involved in cell mediated immune response network in the wild type- or mutant-infected animals. A subset of 15 most highly differentially expressed genes in this network is listed in **(F)**. The plots were drawn with descending level of gene expression sorted in the mutant-infected samples (top to bottom). The scale bar ranges from +2 (red) to −2 (blue) and is common to all panels.

In general, host response triggered during infection and/or injury leads to restriction of damage to the targeted host and/or prevention/recovery from the injury caused by the attack. To determine the host response to infection by wild type or mutant *Cryptococcus*, we interrogated the respective SDEG for their expression pattern and selective role in cell viability and survival, proinflammatory response, and cell-mediated immune response networks (Figure 3 and Additional file 3: Table S3). A subset of 170 SDEG was involved in cell viability and survival network. These genes encode for cytokines (12 genes), enzymes (51 genes), transcriptional regulators (21 genes), transmembrane receptors (23 genes) and transporters (12 genes). Of these SDEG, expression of more than 67% (115 genes) was up-regulated and 55 genes were down-regulated in the mutant- infected mouse brain. (Figure 3A, 3D and Additional file 3: Table S3). In contrast, expression of 45 SDEG was up- and 37 were down-regulated in the wild type-infected mouse brain.

Similarly, of the 174 SDEG involved in the proinflammatory response network, only 51 were up-regulated in the wild type-infected mouse brains, compared to 135 SDEG in the mutant- infected ones (Figure 3B, 3E and Additional file 3: Table S3). In addition, of the 174 SDEG, expression of 24 were down-regulated in the wild type-infected and 39 were down-regulated in the mutant-infected mouse brain, respectively, while other 99 SDEG showed similar expression levels in mice infected by either wild type or the mutant.

The cell-mediated immune response network comprised of a subset of 102 SDEG (Figure 3C, 3F and Additional file 3: Table S3) that encode for cytokines (12 genes), enzymes (17 genes), transcriptional regulators (24) and transmembrane receptors (25 genes). Among these, expression of 83 was up-regulated and 19 were down-regulated in the mutant-infected mouse brain. In contrast, 28 SDEG were up-regulated and 13 were down-regulated in the wild type-infected mouse brain.

Taken together, both the number of SDEG and their expression pattern suggests significant activation of cell viability and survival, proinflammatory response as well as cell-mediated immune response networks/pathways, predominantly in the mutant-infected, compared to the wild type-infected, mouse brain.

### Differential regulation of IFN-γ and TNF-α network in mouse brains infected by the wild type or *itr1aΔ itr3cΔ* double mutant

**Figure 4:**
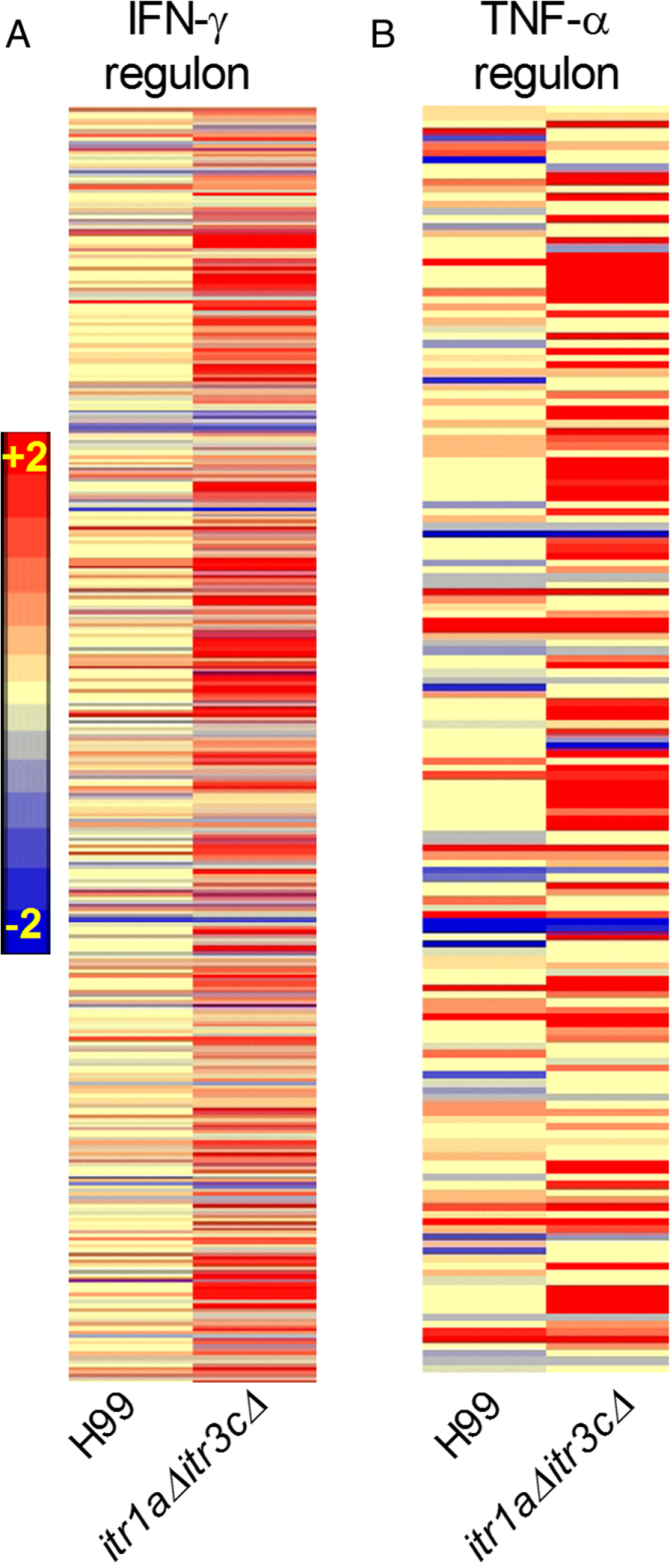
**Expression of genes regulated by IFN-γ and TNF-α. (A)** Intensity plot of SDEG (715 genes) regulated by IFN-γ in the wild type - or the *itr1aΔ itr3cΔ* mutant-infected animals. **(B)** Intensity plot of SDEG (173 genes) regulated by TNF-α in the wild type- or mutant-infected animals. The scale bar ranging from +2 (red) to −2 (blue) in both (A) and (B).

To understand the molecular correlates of differential pathogenesis between the wild type- and mutant- infected mouse brains, we analyzed the expression pattern of SDEG regulated by IFN-γ and TNF-α, two key cytokines that are crucial for the host defense against microbial infection (Figure 4 and Additional file 4: Table S4). There were 715 SDEG in the IFN-γ regulon that encodes for cytokines (21 genes), enzymes (195 genes), transcriptional regulators (69 genes), transmembrane receptors (69 genes) and transporters (49 genes). Of these SDEG, 472 (66%) were up-regulated and 243 (34%) were down-regulated, in the mouse brain infected by the mutant. In contrast, the number of up- and down-regulated SDEG decreased to 182 (25.5%) and 140 (19.5%), respectively, in the wild type-infected mouse brain (Figure 4A).

Of the 173 SDEG regulated by TNF-α, expression of 96 genes were up-regulated and 19 genes were down-regulated in the mutant-infected animals. In contrast, 59 SDEG were up-regulated and 42 were down-regulated in the wild type-infected mouse brain (Figure 4B). Thus, both the number of SDEG involved in the IFN-γ and/or TNF-α regulon and their expression pattern suggest significant activation of the host protective immune networks/pathways in the mutant-infected, compared to the wild type-infected, mouse brain.

### Activation of a selective Th1 response network and the canonical IFN signaling pathway in the mouse brain infected by the *itr1aΔ itr3cΔ* double mutant

**Figure 5:**
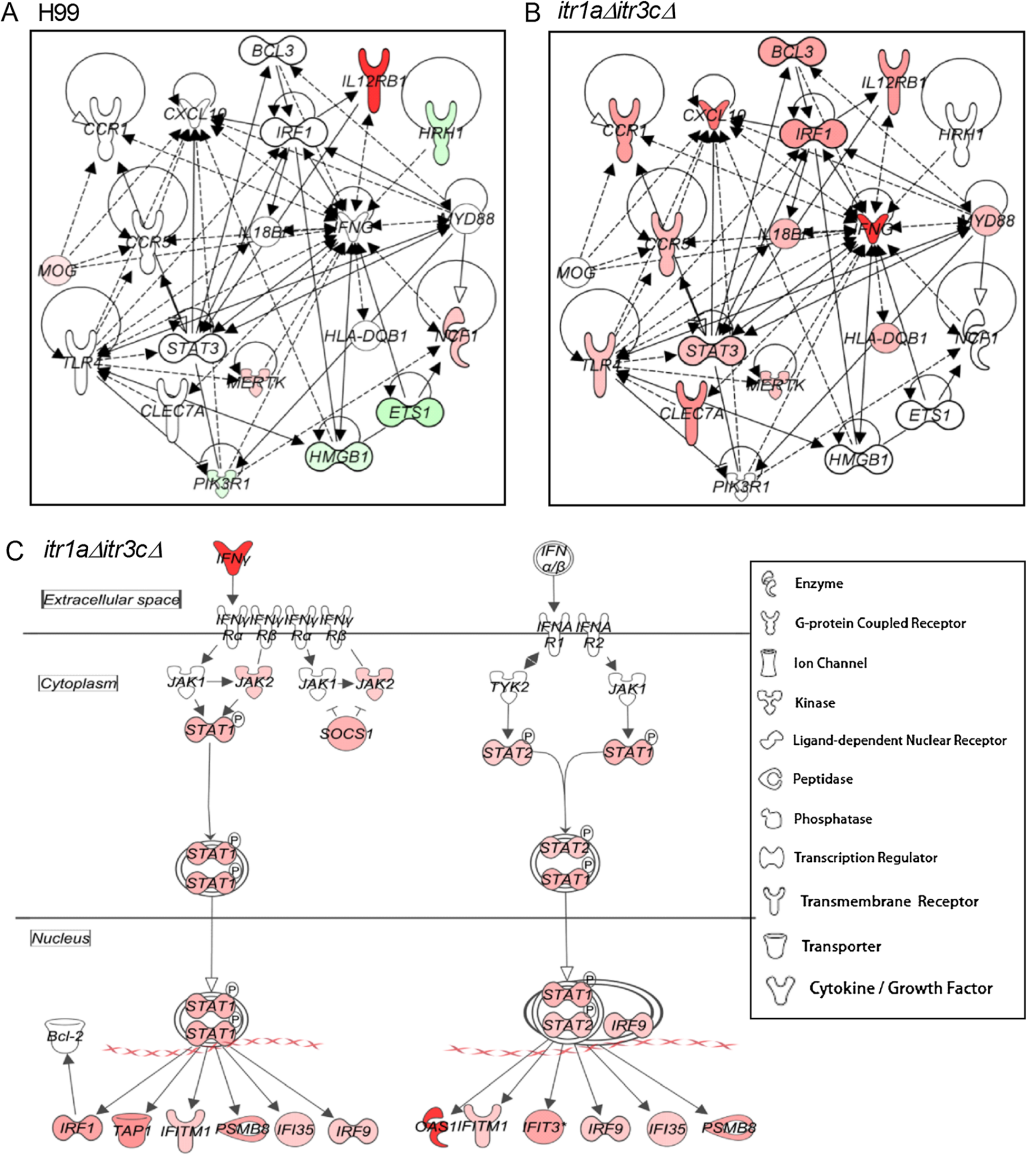
**Expression of genes in the Th1 response network and interferon signaling pathway. (A)** Interaction and expression of Th1 response genes in the wild type H99-infected animals. **(B)** Interaction and expression of Th1 response genes in the *itr1aΔ itr3cΔ* mutant-infected animals. **(C)** Interaction and expression of canonical interferon signaling pathway genes in the mutant-infected animals. Shapes in red are up-regulated and green are down-regulated. Intensity of the shape colors is relative to the level of gene expression (dark color denotes stronger expression). Solid lines represent direct interaction and broken lines show indirect interactions. The legend is common for (A), (B) and (C).

We next interrogated the SDEG for their involvement in the host protective, Th1 response network and canonical interferon (IFN) signaling pathway (Figure 5). Of the 20 SDEG that are part of the Th1 response network, only four each were up- and down-regulated, respectively, in the wild type-infected mouse brains (Figure 5A). In contrast, in the mutant-infected mouse brains, 14 out of 20 SDEG involved in Th1 response, including *Tlr4*, *Ifng*, *Cxcl10*, *Stat3*, *Irf1*, *Ccr5* and *Ccr1* were significantly up-regulated, (Figure 5B). Similarly, expression of several genes in the canonical IFN signaling pathway, including *Ifng*, *Jak2*, *Stat1*, *Stat2*, *Socs1*, *Irf1*, *Irf9*, *Tap1*, *Ifitm1*, *Psmb8*, *Ifi35*, *Gas1* and *Fit3* were up-regulated during infection by the mutant strain (Figure 5C). In contrast, expression of only three genes (*Stat1*, *Tap1* and *Psmb8*) was up-regulated and *Bcl2* was down-regulated in the wild type-infected animals. Taken together, both the number of up-regulated SDEG and their level of expression in the Th1 response network, as well as IFN signaling pathway suggest activation of host protective immune response during infection by the mutant-, compared to the wild type-infected mouse brain.

### Activation of alternate complement activation pathway in mouse brain infected by the *itr1aΔ itr3cΔ* double mutant

**Figure 6:**
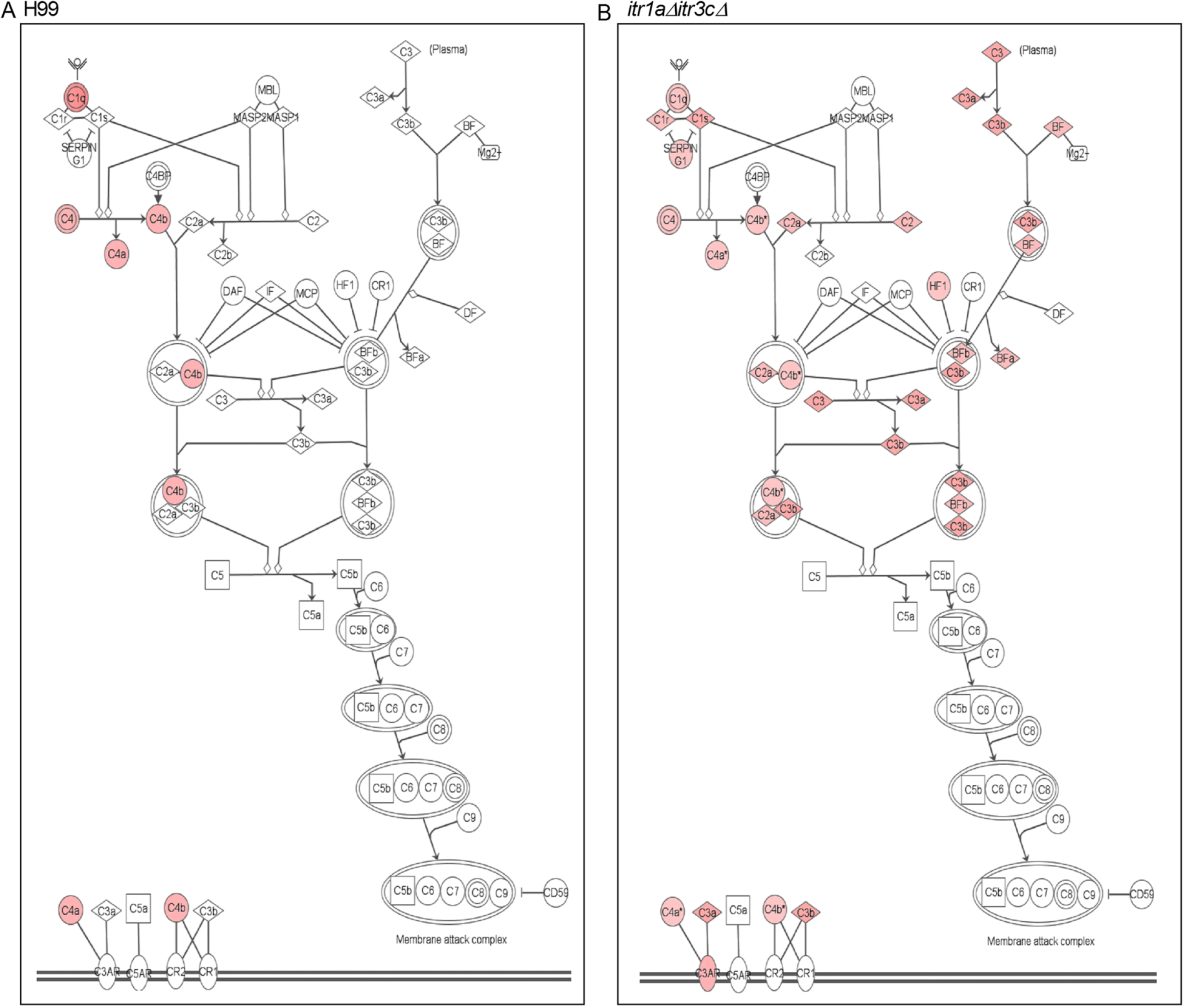
**Expression of genes in the canonical complement system pathway. (A)** Interaction and expression of canonical complement system pathway genes in the wild type H99-infected animals. **(B)** Interaction and expression of canonical complement system pathway genes in the mutant-infected animals. Shapes in red are up-regulated and the intensity of shape colors is relative to the level of gene expression (dark color denotes stronger expression).

Finally, we examined the SDEG to decipher the complement-mediated innate immune response network in the infected mouse brains. Complement activation pathways play crucial role during cryptococcal infection. While complement components, such as C1 (q, r, s), C2a and C4 (a, b) are classified as classical activation pathway, C3 (a, b) and BF (a, b) are considered as members of the alternate activation pathway (Figure 6). Among the SDEG that code for classical pathway, *C1q*, *C4a* and *C4b* were significantly up-regulated both in the wild type and mutant-infected animals (Figure 6A and 6B). However, SDEGs that encode other members of the classical pathway (*C1r*, *C1s* and *SerpinG1*) were up-regulated only in the mutant-infected mouse brain (Figure 6B). Surprisingly, the SDEG involved in the alternate pathway (*C3a*, *C3b*, *Bfa*, *Bfb* and *Hf1*) were significantly up-regulated in the mutant-infected mouse brain; these genes were not significantly affected during wild type-infection (Figure 6A and 6B). In summary, while most genes in the classical complement activation pathway were up-regulated in wild type as well as mutant-infected animals, selected genes in the alternate pathway are predominantly up-regulated in the later, suggesting an efficient innate, complement-mediated protection of the host during infection by this mutant strain.

### Validation of RNA-Seq by qRT-PCR analysis

**Figure 7:**
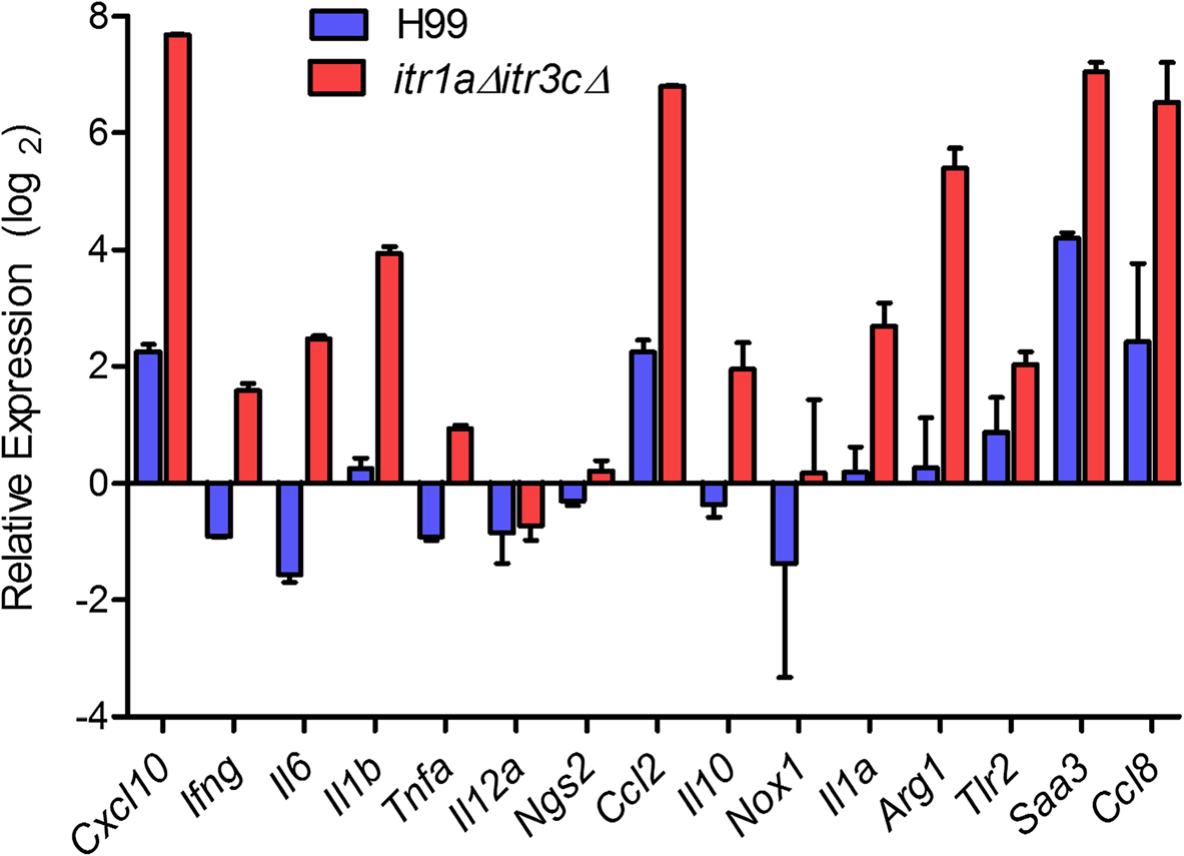
**Gene expression analysis by qRT-PCR.** Expression levels of 15 host genes were measured in the wild type or the *itr1aΔ itr3cΔ* double mutant-infected mouse brains. Gene expression levels are plotted as relative expression (log2) (average ± SD) in each sample, compared to the levels in the uninfected mice brain. The expression level of each gene was normalized to the levels of endogenous *Gapdh* in each of the test and control sample. Gene expression was measured three times in each of the three samples per group.

To confirm and validate the level and pattern of gene expression observed in our RNA-Seq analysis, we performed qRT-PCR analysis. We selected 15 genes randomly to avoid selection-bias and used the same total RNA from RNA-seq experiments as templates for the qRT-PCR. Consistent with the RNA-seq results, expression of *Cxcl10*, *Ifng*, *Il6*, *Il1b*, *Il1α*, *Ccl2*, *Arg1*, *Saa3* and *Ccl8* were up-regulated and *Psap* was down-regulated in the mutant-infected, compared to the wild type-infected mouse brain (Figure 7). Taken together, the pattern and extent of gene expression determined by qRT-PCR analysis is consistent with the RNA-Seq analysis, thus confirming the consensus between these two methodologies and validating our RNA-Seq-based gene expression analysis.

### The *itr1aΔ itr3cΔ* double mutant is defective in cell surface structure

**Figure 8:**
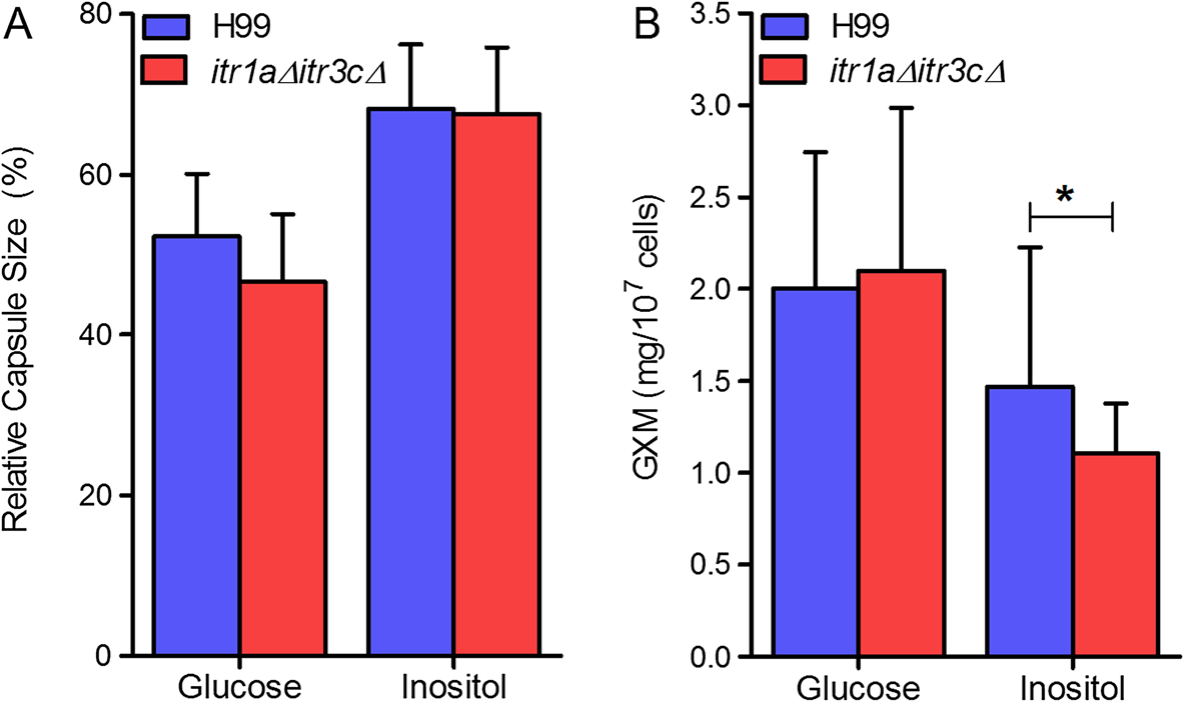
**Capsule size and level of GXM secreted by the wild type and** ***itr1aΔ itr3cΔ*** **mutant strain. (A)** Size of capsule produced by the wild type H99 and the *itr1aΔ itr3cΔ* mutant when grown in glucose or inositol as carbon source. Relative capsule size was determined by capsule size/(capsule size + cell size) in 100 cells. **(B)** Amount of GXM released by fungal cells when grown in glucose or inositol as carbon source. *Statistically significant based on Students’ *t*- test (P < 0.001). Each experiment was repeated at least 3 samples in triplicate.

Based on our previous study, we hypothesized that the difference in fungal cell surface structure between the wild type and *itr1aΔ itr3cΔ* mutant could contribute to the differential host response in the infected mouse brains. We have shown [9] that the *itr1aΔ itr3cΔ* double mutant had reduced production of hyaluronic acid (HA), a ligand required for fungal cell interaction with endothelial cell during BBB crossing [19,20]. In addition, *Cryptococcus* can use inositol as a sole carbon source, in which condition, fungal cells remain encapsulated. This phenomenon indicates that inositol may be utilized to regulate capsule production, especially under the conditions with abundant inositol, such as inside the brain. To test the hypothesis that inositol is required for capsule formation and that mutation of two major fungal inositol transporters (Itr1a and Itr3c) may result in an altered capsule structure, we measured capsule size and GXM secretion in the wild type and *itr1aΔ itr3cΔ* mutant under conditions with different inositol levels. Our results showed that both wild type and mutant cells produced significantly larger capsule (P < 0.0001) when they were cultured on medium with inositol as a carbon source than on medium with glucose as a carbon source (Figure 8A). There was no significant difference observed in the capsule sizes between wild type and mutant cells when grown on either glucose or inositol medium (Figure 8A). In addition, though the capsule size of mutant strains, as measured in the Grocott Methenamine Silver-stained sections of infected mouse brain, was smaller than the wild type counterpart, the difference was not statistically significant (Additional file 5: Figure S1). However, the secretion of GXM was significantly reduced when fungal cells were grown *in vitro* on inositol medium (P < 0.0001). In addition, the GXM secretion by the mutant strain was significantly reduced, compared to the wild type, when inositol was used as a sole carbon source (P < 0.0005) (Figure 8B).

Taken together, our data suggest that activation of the host protective response in mouse brains infected by the *itr1aΔ itr3cΔ* double mutant is associated with its defect in inositol utilization, which may contribute to the *in vivo* alteration of either the capsule structure or GXM secretion or both. However, the potential role of additional factors, including the alteration of HA production and phospholipids observed in the mutant strain [9], remains to be determined *in vivo*.

### Reduced disease pathology in *itr1aΔ itr3cΔ* double mutant infected mouse brain

**Figure 9:**
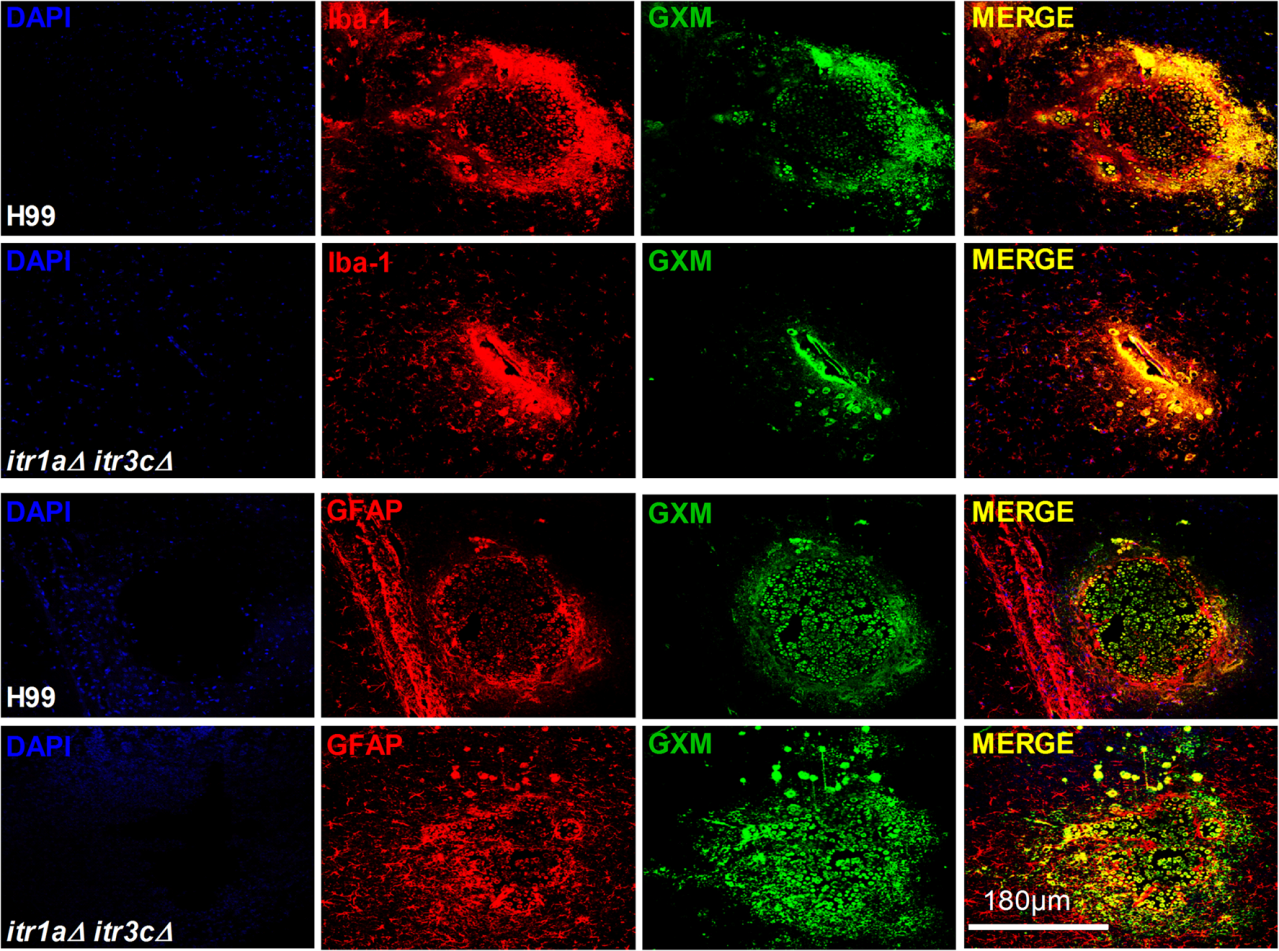
**Immunohistologic analysis of wild type or** ***itr1aΔ itr3cΔ*** **mutant infected mouse brain.** Antibody mediated immunofluorescent staining were performed using brain sections from mouse infected with the wild type H99 or the *itr1aΔ itr3cΔ* mutant strain. The stained sections were analyzed by confocal microscopy followed by 3D reconstructions of images. Tissue sections were stained for the host cell nuclei (DAPI, blue), fungal GXM (FITC-labeled GXM antibody 18B7, Green), host GFAP (astrocyte marker, red) or host Iba-1 (macrophage/microglia marker, red). Scale bar: 180 μm.

To determine the extent of disease pathology elicited by the wild type and mutant strain, we performed immunohistologic staining on the infected mouse brain using antibodies against GXM (a marker for *C. neoformans*), GFAP (an astrocyte marker) or Iba1 (a macrophage/microglia marker). The stained sections were analyzed by confocal microscopy and the images were subsequently reconstructed in x, y and z axis (3D reconstruction) (Figure 9). Consistent with our *in vitro* measurement data (Figure 8), the confocal imaging results showed less GXM staining around the brain lesions infected by the *itr1aΔ itr3cΔ* mutant strain. The brain lesions from wild type-infected mouse had GFAP-positive astrocytes surrounding the fungus inside (Figure 9). Though the mutant-infected brain sections had lesions with the fungus inside, it was not well contained; the GFAP positive cells were not tightly surrounding the lesion, rather they were found diffused. In these samples, more GFAP signal was detected at sites distal to the lesion. Similar results of fungal localization and microglial cell activation were observed in the Iba1 stained brain sections (Figure 9). Taken together, infection of mouse brain by the mutant strain caused a more wide spread activation of astrocytes and microglial cells, which is consistent with our transcriptome data.

## Discussion

Cryptococcosis has emerged as a major fungal infection, mainly due to the increase in population with immune deficiencies caused, for example, by the epidemic of HIV/AIDS and increased use of immunosuppressive drugs. The complex host response to cryptococcal infection involves innate cellular and humoral immune responses as well as cell-mediated adaptive immune responses [21-23]. The antifungal activity of alveolar macrophages as the first line of host defense mechanism against cryptococcal infection has been a subject of intensive investigation [24-26]. Similarly, the relative roles of complement components, surfactant molecules and innate cellular responses during *Cryptococcus*-host cell interaction have been described earlier [21,22]. Moreover, the importance of CD4^+^ and CD8^+^ T cells in eliciting host immune response during fungal infections are well recognized. These studies, as well as several others on the role of various cytokines and their expression profiles during cryptococcal infection, have consistently showed that a protective immune response is closely associated with a robust Th1-type immunity [27,28].

We have recently shown that interaction between the fungal inositol transporters and the host inositol is required for the development of cryptococcosis in murine models. Despite the fact that mice infected by wild type and the *itr1aΔ itr3cΔ* double mutant showed similar *in vivo* growth rates in brain, a delayed killing of those infected by the mutant, compared to the wild type, was observed in a mouse intracerebral infection model [9]. This led to our current hypothesis that infection by the mutant strain may trigger different and/or increased protective host immune responses. Consistently, our data showed a clear pattern of greater activation of protective immune response during infection by the mutant, compared to wild type strain. Moreover, dampening of the network gene expression involved in host detrimental processes such as necrosis and cell death that are associated with inflammation, supports our hypothesis that the host is more tolerant to the infection by the mutant compared to the wild type. Because of the similar *in vivo* growth of the wild type and the *itr1aΔ itr3cΔ* mutant inside the infected brains, the degree of difference in the host defense response may not be enough to cause an increased killing of fungal cells, which is consistent with our data showing a modest prolonged survival rate of mice infected by the mutant, compared to the wild type [18]. In the present study, differential gene expression profile in the wild type- and mutant-infected mouse brain is a net result of differential expression of all the cells at the site of infection (both resident and/or recruited during to infection). However, the nature and distribution of various immune cell populations in the mouse brain infected by the wild type or mutant strains and their role in differential pathogenesis are unknown and are currently under investigation.

In our study, the exacerbated activation of neurological diseases and cell death network genes in the brain is associated with the early death of mice infected with the wild type strain, relative to the mutant [18]. Activation of host cell destruction networks/pathways is corroborated by our histological observation that showed extensive disease pathology in these chronically-infected mice brains. In contrast, infection by the mutant strain, which is attenuated for virulence, is associated with significantly elevated expression and activation of host genes involved in host protective immunity networks/pathways. Specifically, genes involved in cell viability and survival, proinflammatory response, cell mediated immunity and complement-receptor (CR) mediated phagocytosis, as well as IFN-γ and TNF-α regulon network were significantly activated in the mutant-, compared to wild type- infected, mouse brains. During cryptococcal infection, engagement of surface receptors, such as TLR-4, has been reported to signal TNF-α production, activation of macrophages and dendritic cells as well as other immune cells that are important not only for the early control of infection, but also essential for IFN-γ production by the T-cells and establishing an effective adaptive immunity [29]. Together, IFN-γ and TNF-α regulate several downstream cellular networks/pathways involved in the host protective Th-1 type immune response against infecting pathogen [30,31]. This is evident by the increased cryptococcosis among humans and experimental animals that are either defective in producing these cytokines or lacking sufficient T cells [32,33]. In fact, IFN-γ has been shown to help eliminate *Cryptococci* from the CSF [34]. In addition, mice inoculated with recombinant *C. neoformans* expressing murine IFN-γ were protected from subsequent challenge with a virulent strain [35]. Moreover, mice vaccinated with either heat-killed *Cryptococcus* cells or culture filtrate antigens showed significant T-cell-dependent delayed-type hypersensitivity reaction and were better protected against subsequent challenge with a virulent strain [36,37]. Similar to these studies on animal models, elevated IFN-γ levels in the CSF, as well as administration of recombinant IFN-γ were directly correlated with better protective response against cryptococcal infection in humans [38]. Taken together, these findings are consistent with and corroborated by our gene expression analysis that showed an association between activation of the Th-1 type immune networks/pathways and improved host protection.

We noted significant activation of canonical complement activation by alternative pathway, in the brains of mutant- but not wild type-infected mice. The role of complement system in the protection against cryptococcal infection has been well established [21,22]. Guinea pigs depleted of complement components C3-C9 prior to cryptococcal infection showed higher mortality than the untreated animals [39]. Importantly, the outer capsule of *Cryptococcus sp.* has been shown to bind with C3, a component of the alternate complement system and activates the respective pathway *in vitro* [40-42]. However, binding of C3, and thus the alternating complement activation cascade, is largely dependent on the nature and density of the capsule. Therefore, any alteration in the capsule content or thickness impacts the host-pathogen interactions during cryptococcal infection [42-44]. Since complement activation ultimately culminates in the onset of effective host innate immune responses, we suggest that the differential expression of genes involved in various complement activation pathways elicited by the wild type and the *itr1aΔ itr3cΔ* mutant likely contributes to a corresponding difference in their recognition by the host phagocytes. However, further experiments are warranted before concluding that differences in complement mediated phagocytosis of the wild type and mutant strains is a factor for the differential immune response elicited by these two strains.

To determine the molecular immunologic correlates of altered host protective immune response in infected mouse brain, we hypothesized that the *itr1aΔ itr3cΔ* mutant has a different cell surface structure than the wild type. Previously we have shown a significant difference in fungal HA and phospholipid production between the wild type and mutant [9], which may contribute to their differential host response during brain infection. Since that inositol can regulate the production of fungal capsule under conditions of abundant inositol availability, and that the capsule can modulate host immunity, we focused our current study on the capsule production and structure. We analyzed the capsule size and measured the secretion of GXM polysaccharide in wild type and the *itr1aΔ itr3cΔ* mutant *in vitro*. Polysaccharide and its release *in vivo* were also detected by immunohistologic staining of mouse brain using a GXM-specific monoclonal antibody. We observed a modest reduction in the average capsule size between these two cryptococcal strains. Importantly, the GXM secretion was significantly reduced in the mutant, compared to the wild type in the inositol medium, which could contribute to the difference in respective host immune responses in infected mouse brain. Capsule has been shown to suppress the host protective immune response [45,46]. In addition, secretion of GXM has been shown to cause the disruption of BBB during fungal meningitis, indicating the importance of GXM secretion in fungal-host interaction [8]. Therefore, we propose that the difference in cell surface structure, especially in polysaccharide production, contribute to an altered host response, which results in a prolonged survival of mice infected with the mutant compared to the wild type strain. However, at present, we cannot rule out the contribution of additional fungal factors regulated by inositol to the differences in host immune responses. It is also possible that inositol utilization by the fungal cells may regulate the lipid composition and secretion of certain host effectors that could lead to altered host response during CNS infection. Based on our findings, we propose that the modest difference in the cell surface structure observed between the wild type and the *itr1aΔ itr3cΔ* mutant cells may be sufficient to tip the immune balance yet not enough to impact the survival of the fungal cells in the infected brain.

## Methods

### Ethics Statement

The animal studies conducted at Rutgers University were in full compliance with all of the guidelines set forth by the Institutional Animal Care and Use Committee (IACUC) and in full compliance with the United States Animal Welfare Act (Public Law 98–198). The Rutgers IACUCs approved all of the vertebrate studies. The studies were conducted in facilities accredited by the Association for Assessment and Accreditation of Laboratory Animal Care (AAALAC).

### Murine infection and RNA preparation

*C. neoformans* wild type strain and the *itr1aΔ itr3cΔ* double mutant were grown at 30°C on yeast extract-peptone-dextrose (YPD) agar medium. For animal infection, *Cryptococcus* cells were grown at 30°C overnight and cultures were washed twice with 1x phosphate-buffered saline (PBS) by centrifugation, and resuspended at a final concentration of 2 × 10^5^ CFU/ml. Groups of three female A/Jcr mice (NCI-Frederick, MD) were used for each infection. For intravenous injection model, 5 × 10^4^ yeast cells in 100 μl volume for each strain were inoculated *via* tail vein injection. Animals that appeared moribund or in pain were sacrificed by exposing to CO_2_.

Total RNAs was isolated from mouse brains that were either infected by the wild type or the *itr1aΔ itr3cΔ* mutant (n = 3) for 7 days. A group of 3 mice injected with PBS buffer were used as uninfected control. Each brain samples were processed separately for total RNA isolation using Trizol reagent, treated with DNase I and purified with RNA clean-up kit (Clontech, Mountain View, CA). Purified total RNAs from the infected and uninfected mouse brains were quantified using a Nanodrop spectrometer (Thermo Scientific, Waltham, MA) and used in the RNA-Seq and qRT-PCR experiments. The mRNA was enriched by using the oligo(dT) magnetic beads and disrupted to shorter fragments (about 200 bp). The first strand cDNA was synthesized by random hexamer-primer using the mRNA fragments as templates. The double strand cDNA was purified with QiaQuick PCR extraction kit and washed with EB buffer for end repair and single nucleotide A (adenine) addition. Finally, sequencing adaptors were ligated to the fragments. The required fragments were gel purified and enriched by PCR amplification. The library products were used for sequencing analysis via Illumina HiSeq™ 2000 at BGI (Shenzhen, China).

### RNA-Seq Data analysis

The gene expression level was calculated by using RPKM method (Reads Per Kb per Million reads) as described [47]. The log2-transformed transcript levels from uninfected, wild type-infected or mutant-infected mice brain (n = 3 per group) was further analyzed by Partek Genomics Suite (Partek, St.Louis, MO) for statistical analysis and to identify significantly differentially expressed genes (SDEG) in the infected group, relative to the uninfected animals. The normalized RNA-Seq data from each group (n = 3) were averaged and analyzed by one-way ANOVA with equal variance for significance [48]. To identify SDEG, the log2 ratio between uninfected and wild type- or the mutant-infected transcript levels was further screened by applying an unadjusted false discovery rate (FDR) of 5% (q value ≤ 0.05). The lists of SDEG for wild type- or mutant-infected mice brains were further used to decipher the GO and network/pathway analysis.

### Network/Pathway analysis

We used Ingenuity Pathway Analysis (IPA) program (Ingenuity Systems, Redwood, CA) to determine the gene ontology, biological functions, networks and pathways that were perturbed by the SDEG from wild type- or mutant-infected animals. Gene ontology analysis was performed by interrogating the SDEG generated in our studies with the IPA knowledgebase as described [48]. To improve the stringency, we included only mouse database from IPA for the network/pathway analysis. We used a z-score algorithm from IPA to predict the biological functions that are significantly affected by the SDEG. The z-score algorithm in IPA predicts the activation status of a biological function based on the direction and pattern of expression of member genes. A z-score of ≥ +2 indicates activation, while ≤ −2 refers inhibition of a network/pathway in a biological function. For regulon analysis, we included all the interacting partners (both directly and indirectly) of regulator genes, *Ifng* or *Tnfa*, from the SDEG dataset. These regulon are comprised of enriched genes that are either regulating or regulated by IFN-γ or TNF-α*.*

### qRT-PCR analysis

Purified total RNA from the infected and uninfected mouse brains used for RNA-seq was also used as templates for cDNA synthesis for qRT-PCR assay. The qRT-PCR experiments were performed using SYBR advantage QPCR premix reagents (Clontech, Mountain View, CA) in a MxPro3005P qPCR system as per the manufacturers’ instructions (Agilent Technologies, Santa Clara, CA). Expression level of *Gapdh* was used as control to normalize the value across different target genes. The threshold cycle (Ct) values for individual genes were calculated by using MxPro Software (Agilent Technologies, Santa Clara, CA) and the transcript abundance was calculated using the formula 2^∆Ct^, where ∆Ct refers to the change in Ct between the target gene and *Gapdh*. Student’s t-test was used to calculate the *p*-value significance for the differential gene expression between infected and uninfected samples.

### Measurement of capsule and GXM in *Cryptococcus*

*C. neoformans* H99 (wild type) or the *itr1aΔ itr3cΔ* double mutant cells (10^9^) were inoculated into 250-ml Erlenmeyer flasks containing 60 ml of minimal medium [49] supplemented with glucose or inositol as carbon source. Fungal cells were cultivated for 7 days at 30°C with shaking (220 rpm). Then the capsule size and cell size was measured after India ink staining. More than 100 cells for each condition were measured each time. The relative capsule size was calculated using the following formula: relative capsule size (%) = 100*capsule diameter/(cell diameter + capsule diameter). The GXM isolation and measurement were followed the protocol described by Wozniak and Levitz [50]. Each experiment was repeated at least three times. Statistical analysis was performed by two tailed student t-tests. P <0.001 indicates statistical significance.

### Immunofluorescence and confocal analysis

Brain tissue sections were fixed in formalin and embedded in paraffin. After de-paraffinization, tissue sections were cut to 20 μm and were incubated in blocking solution (5 mM EDTA, 1% fish gelatin, 1% essentially Ig-free BSA, 2% human serum and 2% horse serum) for 3 hours at room temperature. Tissue sections were incubated with the primary antibody (anti-GXM, anti-GFAP, 1:100, or anti-Iba-1, 1:400) overnight at 4°C. Samples were washed several times with PBS at room temperature and incubated with appropriate secondary antibodies conjugated to FITC or Cy3 for 1 hour at room temperature, followed by another wash in PBS for 1 hour. Tissue sections were then mounted using antifade reagent with DAPI (Life Technologies, Grand Island, NY) and the cells were examined in a A1 confocal microscope (Nikon, Melville, NY). Antibody specificity was confirmed by replacing the primary antibody with a non-specific myeloma protein of the same isotype or non-immune serum.

## Additional files

Additional file 1: Table S1. Quality assessment of RNA-Seq data from the wild type or *itr1aΔ* *itr3cΔ* mutant infected mouse brain. Additional file 2: Table S2. Expression level of SDEG involved in neurological disorder and cell death networks in the wild type or the *itr1aΔ itr3cΔ* mutant infected mouse brain. Additional file 3: Table S3. Expression level of SDEG involved in cell viability and survival, proinflammatory response and cell mediated immunity networks in the wild type or the *itr1aΔ itr3cΔ* mutant infected mouse brain. Additional file 4: Table S4. Expression level of SDEG involved in IFN-g and TNF-a regulon in the wild type or the *itr1aΔ itr3cΔ* mutant infected mouse brain. Additional file 5: Figure S1. Capsule of wild type and *itr1aΔ itr3cΔ* mutant strains. (A) Relative capsule size of wild type and *itr1aΔ itr3cΔ* mutant cryptococcal cells. (B) Representative image of wild type H99 cell in infected mouse brain stained with Grocott Methenamine Silver. (C) Representative image of *itr1aΔ itr3cΔ* mutant cell in infected mouse brain stained with Grocott Methenamine Silver.
