# Supplementary material for: Cryptococcus inositol utilization modulates the host protective immune response during brain infection
Source: Cell Commun Signal. 2014 Sep 10;12:51. doi: 10.1186/s12964-014-0051-0 (PMC4172957; doi:10.1186/s12964-014-0051-0)
Supplement: Additional file 1: Table S1. — Quality assessment of RNA-Seq data from the wild type or itr1aΔ itr3cΔ mutant infected mouse brain. [file 12964_2014_51_MOESM1_ESM.doc]

| RNA samples* | Total basepairs | Total Reads | Rate of Clean Reads | Coverage to genome (%) |
| --- | --- | --- | --- | --- |
| Uninfected | 343,566,244 | 7,011,556 | 99.29 | 88.55 |
| Wild type H99 infected | 344,302,567 | 7,026,583 | 99.18 | 87.74 |
| *itr1a∆ itr3c∆* infected | 343,672,770 | 7,013,730 | 99.28 | 88.69 |

**Supplementary Table 1.**  Quality assessment of RNA-Seq data from the wild type or *itr1aΔ itr3cΔ* mutant infected mouse brain

* Total RNA was isolated from uninfected or infected mouse brain (n=3 per group)
