# Supplementary figures and images for: Cryptococcus inositol utilization modulates the host protective immune response during brain infection
Source: Cell Commun Signal. 2014 Sep 10;12:51. doi: 10.1186/s12964-014-0051-0 (PMC4172957; doi:10.1186/s12964-014-0051-0)

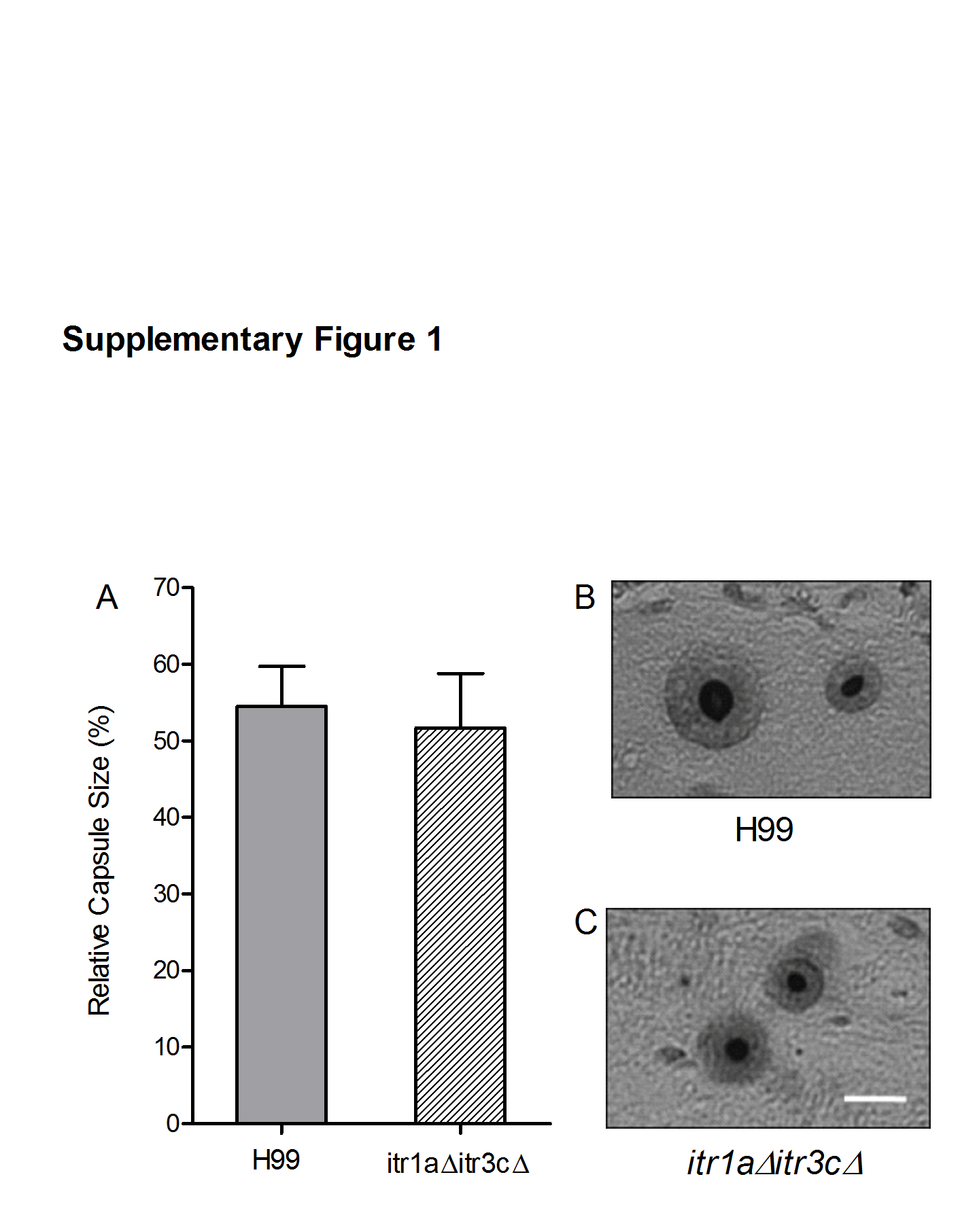

Supplement: Additional file 5: Figure S1. — Capsule of wild type and itr1aΔ itr3cΔ mutant strains. (A) Relative capsule size of wild type and itr1aΔ itr3cΔ mutant cryptococcal cells. (B) Representative image of wild type H99 cell in infected mouse brain stained with Grocott Methenamine Silver. (C) Representative image of itr1aΔ itr3cΔ mutant cell in infected mouse brain stained with Grocott Methenamine Silver. [file 12964_2014_51_MOESM5_ESM.tiff]
